# The potential protective effects of probiotics, prebiotics, or yogurt on chronic obstructive pulmonary disease: Results from NHANES 2007–2012

**DOI:** 10.1002/fsn3.4332

**Published:** 2024-07-17

**Authors:** Yu Hong, Ting Luo

**Affiliations:** ^1^ Department of Pulmonary and Critical Care Medicine Ninth People's Hospital of Chongqing Chongqing China

**Keywords:** COPD, NHANES, prebiotics, probiotics, yogurt

## Abstract

Chronic obstructive pulmonary disease (COPD) ranks among the world's three leading causes of mortality, owing to the increased smokers and aging populations. Previous studies showed that gut microbiota could help to ameliorate respiratory diseases. Hence, the current study aims to evaluate the effects of probiotics, prebiotics, or yogurt on the prevalence of COPD. A cross‐sectional study was carried out by investigating data from three consecutive NHANES cycles during 2007–2012. Individuals who met the inclusion and exclusion criteria were studied. Prescription medications and dietary were reviewed to identify the intake of probiotics, prebiotics, or yogurt. The included participants were then divided into two groups depending on their consumption of probiotics, prebiotics, or yogurt. Multivariate logistic regression analysis was conducted to analyze the effects of probiotics, prebiotics, or yogurt consumption on the prevalence of COPD. Out of 7486 enrolled participants, 1656 (22.12%) were categorized into the probiotics, prebiotics, or yogurt intake group. This study indicated that consuming probiotics, prebiotics, or yogurt were correlated with a lower incidence of COPD, even when factors like gender, age, education, moderate‐intensity activities, vigorous activities, hypertension, asthma, diabetes, smoking and alcohol consumption were accounted for (Model 1: OR, 0.68, 95% CI, 0.53–0.87; Model 2: OR, 0.77, 95% CI, 0.59–0.99; Model 3: OR, 0.75, 95% CI, 0.57–0.98). The findings reveal that consuming probiotics, prebiotics, or yogurt might play a beneficial role in preventing COPD.

## Graphical abstract

A comprehensive cross‐sectional study was carried out on a representative group of 7486 individuals in the US, revealing that consuming probiotics, prebiotics, or yogurt was correlated with a 25% reduction in the incidence of chronic obstructive pulmonary disease (COPD). The finding suggests that altering intestine microbiota potentially helps to prevent the occurrence of COPD.

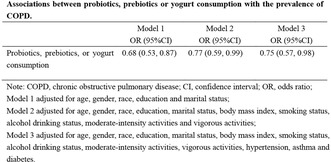

## 1 INTRODUCTION

Chronic obstructive pulmonary disease (COPD) manifests as a diverse pulmonary condition, marked by persistent respiratory issues (such as coughing, sputum production, shortness of breath, and flare‐ups), stemming from airway and alveoli irregularities leading to permanent blockage of airflow (Celli et al., 2022). Currently, COPD ranks among the world's three leading causes of mortality, with 90% of these deaths happening in developing countries, which causes a substantial and increasing economic and social burden (Halpin et al., 2019; Meghji et al., 2021; Stolbrink et al., 2022). It indicates that the rising smoking rates in developing countries, along with the aging demographic in developed countries, will lead to upwards of 5.4 million yearly fatalities due to COPD and similar ailments by 2060 (World Health Organization, 2022).

COPD emerges from intricate interactions between genes and the environment throughout one's life (Agustí et al., 2022). Chronic inflammation is a key factor in the emergence of COPD, which causes remodeling and narrowing of the small airways and destruction of the lung parenchyma, consequently resulting in progressive airflow limitation (Barnes, 2016). Recent researches suggest that gut microbiota might affect the lung's mucosal immune response and control chronic inflammation, playing a role in the development of COPD (Ananya et al., 2021). The interaction between intestinal and pulmonary microbes is known as the “gut‐lung axis.” This axis aids in the transfer of microbial metabolites, hormones, endotoxins, and cytokines into the circulatory system linking the intestine and lung (Budden et al., 2019). The use of antibiotics, exposure to cigarettes and biomass, air pollution, diet changes, and some genetic factors can alternate the distribution of intestinal microbiota (Dang & Marsland, 2019). Despite their anatomical differences, the intestine and lung both stem from endosome and plentiful bacterial populations (Mjösberg & Rao, 2018). This gut dysbiosis is linked to irregularities in lung microbiota and inflammatory conditions in the airways (Ananya et al., 2021). It has been reported that gut microbiota disturbance significantly affected cigarette smoking‐induced COPD development, and fecal microbiota transplantation might ameliorate COPD pathogenesis by inhibiting lung inflammation (Lai et al., 2022). Additionally, some studies also showed a correlation between acute exacerbation of COPD and intestinal microbes' disturbance (Siva et al., 2013; Sun et al., 2020).

Evidence suggest that regulating gut microbiota and its metabolites can either prevent or mitigate respiratory illnesses (Du et al., 2019; Luoto et al., 2014; Wang et al., 2022). These ways include supplementation of probiotics, prebiotics, or yogurt. Probiotics refer to “live microorganisms that when administered in sufficient quantities confer a health benefit on the host” (Marco et al., 2021). Prebiotics are the specific substrates used by host microbes (probiotics) that offer health benefit (Marco et al., 2021). Yogurt always contains abundant probiotics or prebiotics, making it the predominant dietary source for probiotics. Animal studies and in vitro studies revealed that supplementation with probiotics and/or prebiotics could prevent respiratory injury of COPD (Aimbire et al., 2019; Qu et al., 2022). One clinical trial showed that probiotics improved pulmonary symptoms and pulmonary function in COPD patients (Panahi et al., 2017). However, large‐scale studies are lacking to explore the effects of probiotics, prebiotics, or yogurt on the prevalence of COPD. Hence, the objective of our research is to analyze the correlation between the intake of probiotics, prebiotics, or yogurt and the prevalence of COPD by investigating data from National health and Nutrition Examination Survey (NHANES, 2007–2012).

## 2 METHODS

### 2.1 Study population

**FIGURE 1:**
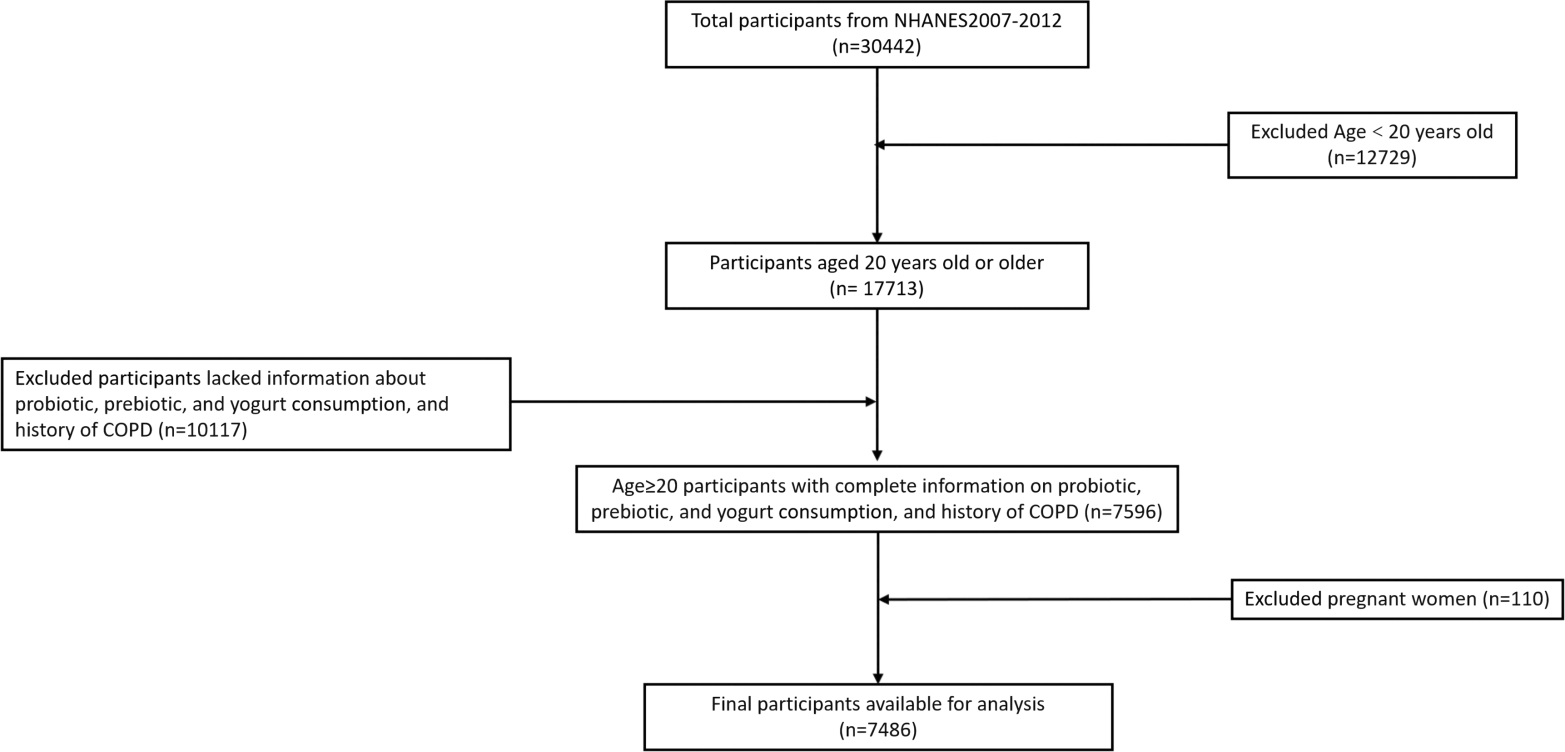
Flow diagram of study participants. COPD, chronic obstructive pulmonary disease; NHANES, National Health and Nutrition Examination Survey.

NHANES represents a cross‐sectional study focusing on civilian population in the U.S. Demographic, dietary, anthropometric, laboratory, and questionnaire information were gathered to evaluate the nutrition and health status of participants (https://www.cdc.gov/nchs/nhanes/about_nhanes.htm) (National center for health statistics, n.d.‐a). The research was approved by the Research Ethics Review Board of the National Centre for Health Statistics, and all participants in NHANES gave their informed consent (https://www.cdc.gov/nchs/nhanes/irba98.htm) (National center for health statistics, n.d.‐b). This study investigated data from three consecutive NHANES cycles during 2007–2012. Initially, a total of 30,442 participants were searched from the NHANES. Then, individuals meeting the following criteria were excluded: (1) age <20 years old (12,729 participants); (2) with missing information on the diagnosis of COPD (three participants) and the intake of probiotics, prebiotics, or yogurt (10,114 participants); (3) pregnant women (110 participants). Finally, 7486 individuals were included in this study. Figure 1 shows the flowchart of the study population.

### 2.2 Diagnosis of COPD

Diagnosis of COPD was confirmed by fulfilling any of these specified criteria (Han & Wang, 2023): (1) after bronchodilation, the FEV1‐to‐FVC ratio was less than 0.7; (2) individuals reported COPD, chronic bronchitis, or emphysema; (3) those over 40 years old with a background of bronchitis or smoking, using medications like inhaled corticosteroids, selective phosphodiesterase‐4 inhibitors, mast cell stabilizers, or leukotriene modifying agents.

### 2.3 Assessment and definition of probiotics, prebiotics, or yogurt consumption

In this study, probiotics, prebiotics, or yogurt supplements were collected in all NHANES year cycles from 2007 to 2012. Since the FDA currently does not regulate probiotics, they are solely accessible as dietary supplements. But there are FDA‐regulated prebiotics products (e.g., *lactulose*) (https://www.accessdata.fda.gov/scripts/cder/daf/index.cfm) (Food and Drug Administration, 2020). Supplementary Table S1 displays a previous list for pinpointing products containing prebiotics and probiotics (O'Connor et al., 2021). Additionally, these questionnaires, such as the dietary interview—first day (the 24‐h dietary recall interview before the survey), the dietary interview—second day (the second 24‐h dietary recall interview collected by telephone 3–10 days after the first interview), and the dietary supplement use—30th day, were used to assess probiotics, prebiotics, and yogurt consumption. In detail, text‐mined for key phrases was used to identify prebiotics, probiotics, or yogurt, which included dietary supplement names and ingredients, and medication names and ingredients. Subsequently, the included participants were divided into two groups depending on their intake of probiotics, prebiotics, or yogurt: abstaining from probiotics, or prebiotics, or yogurt, and consuming probiotics, or prebiotics, or yogurt.

### 2.4 Demographic and other covariates

Age groups were divided into 20–39 years, 40–60 years, and over 60 years. Self‐reported Hispanic origin and race were classified into categories such as Mexican American, non‐Hispanic black, non‐Hispanic white, other Hispanic, and various other races. The calculation of poverty‐to‐income ratio (PIR) depended on the income of the respondent's household in comparison to the federal poverty threshold set for their household size. Individuals with a PIR of 1.35 or less were classified under low income, while those with a PIR of 1.35 or more fell into the middle (1.3–1.85) or high income (≥1.85). Education was divided as either below high school, high school or GED, or university degree or higher. Marital status was segmented into widowed/divorced/separated, married or single.

Body mass index (BMI) is an internationally used measure of obesity and health, calculated from weight and height. BMI was classified into three categories: normal weight (18.5–24.9 kg/m^2^), overweight (25–29.9 kg/m^2^), and obese (≥30 kg/m^2^). Smoking status was segmented into active smokers, former smokers, and those who never smoked (Han & Wang, 2023). It was verified that active smokers engaged in smoking daily, or on certain days. Former smokers were verified by consuming a minimum of 100 cigarettes in their lifetime, yet they were not active smokers. Participants who smoked less than 100 cigarettes throughout their lives were classified as nonsmokers. Alcohol consumption was marked as yes or no for individuals consuming at least 12 alcoholic drinks annually (Yang et al., 2022). Activities of moderate intensity were characterized by causing slight perspiration or a minor to moderate elevation in respiration or heart rate (HR). Vigorous activities were characterized by the induction of intense perspiration or significant elevations in respiration or HR (Karanth et al., 2021).

Diabetes was determined by the following criteria as previously described (Han & Wang, 2023): diabetes self‐reporting; antidiabetic medication usage; glycohemoglobin HbA1c exceeding 6.5%; fasting glucose levels of no less than 7.0 mmol/L; random blood glucose levels of at least 11.1 mmol/L; OGTT blood glucose levels of no less than 11.1 mmol/L. Additionally, individuals exhibiting both impaired fasting glucose and reduced glucose tolerance were grouped together and categorized as borderline diabetes. Hypertension was characterized by an average systolic blood pressure of 140 mmHg or more, a diastolic blood pressure of 90 mmHg or more, or the use of hypertension medication or notification of a hypertension diagnosis by a medical professional (Han & Wang, 2023). Asthma was evaluated through individuals' self‐reports “In the past 12 months have you been told by a doctor or other healthcare professional that you have asthma?”

### 2.5 Statistical analysis

In the descriptive analysis, continuous variables were presented as mean ± standard deviation (SD), and categorical variables were presented in frequency or percentage. Analysis of variance (ANOVA, for normal distribution) or Kruskal–Wallis H (for skewed distribution) was used to compare continuous variables among groups. Chi‐squared test or Fisher's exact test was employed to compare categorical variables among groups. Multivariate logistic regression analysis was conducted by using a generalized linear model to evaluate the correlation between probiotics, prebiotics, or yogurt consumption and the prevalence of COPD. Model 1 was controlled for age, gender, race, education, and marital status. Model 2 was further controlled for BMI, drinking, smoking, moderate‐intensity activities, and vigorous activities based on Model 1. Model 3 further adjusted hypertension, diabetes, and asthma based on Model 2. The regression models included these covariates, relying on prior studies or a variation exceeding 10% in effect estimations. Additionally, to verify if associations varied by demographic factors, this study conducted analyses stratified by age, gender, race, BMI, and asthma and then explored the interaction analyses. Data were analyzed by using the statistical packages R (The R foundation; version 4.2.0) and EmopwerStats (www.empowerstats.net, X&Y solutions, Inc. Boston, Massachusetts). Statistical significance was confirmed as *p‐*values < .05 in a two‐sided test.

## 3 RESULTS

### 3.1 Baseline characteristics of the study population

This study included 7486 participants, of whom 1656 (22.12%) participants were categorized into the probiotics, prebiotics, or yogurt intake group. Demographic characteristics are presented in Table 1. The study found that individuals consuming probiotics, prebiotics, or yogurt tended to be female, youngster (<60 years old), non‐Hispanic white, and possessed greater income and education. Regarding lifestyle habits, individuals consuming probiotics, prebiotics, or yogurt showed a reduced tendency to smoke, engaged in more moderate and intense activities, had a lower incidence of obesity or overweight, and had less diabetes, hypertension, COPD, in contrast to those not consuming probiotics, prebiotics, or yogurt.

**TABLE 1:** Characteristics of US adults according to probiotics, prebiotics, or yogurt consumption: NHANES, 2007–2012.

Characteristics	No probiotics, prebiotics, or yogurt consumption (*n* = 5830)	Probiotics, prebiotics, or yogurt consumption (*n* = 1656)	*p*‐Value
Age (years)			.038
20–39	1383 (23.72%)	398 (24.03%)	
40–59	1878 (32.21%)	582 (35.14%)	
≥60	2569 (44.07%)	676 (40.82%)	
Gender			<.001
Male	2752 (47.20%)	581 (35.08%)	
Female	3078 (52.80%)	1075 (64.92%)	
Race/ethnicity			<.001
Non‐Hispanic White	3092 (53.04%)	963 (58.15%)	
Non‐Hispanic Black	1141 (19.57%)	232 (14.01%)	
Mexican American	672 (11.53%)	154 (9.30%)	
Other Hispanic	514 (8.82%)	153 (9.24%)	
Other race	411 (7.05%)	154 (9.30%)	
Education			<.001
Less than high school	1258 (21.60%)	246 (14.87%)	
High school	1366 (23.46%)	281 (16.99%)	
More than high school	3199 (54.94%)	1127 (68.14%)	
Poverty‐to‐income ratio			<.001
≤1.35	1551 (28.91%)	300 (19.43%)	
1.35–1.85	658 (12.26%)	139 (9.00%)	
≥1.85	3156 (58.83%)	1105 (71.57%)	
Marital status			.058
Single	825 (14.16%)	218 (13.17%)	
Married	3516 (60.34%)	1052 (63.56%)	
Widowed/divorced/separated	1486 (25.50%)	385 (23.26%)	
BMI (kg/m^2^)			.001
18.5–24.9	1493 (25.95%)	495 (30.16%)	
25–29.9	1962 (34.10%)	552 (33.64%)	
≥30	2216 (38.52%)	564 (34.37%)	
Smoking status			<.001
Never	3045 (52.23%)	1022 (61.75%)	
Former	1737 (29.79%)	494 (29.85%)	
Active	1048 (17.98%)	139 (8.40%)	
Alcohol drinking status			.385
Yes	3980 (72.59%)	1120 (71.47%)	
No	1503 (27.41%)	447 (28.53%)	
Moderate‐intensity activities			<.001
Yes	2446 (41.96%)	912 (55.07%)	
No	3383 (58.04%)	744 (44.93%)	
Vigorous activities			<.001
Yes	1107 (18.99%)	444 (26.81%)	
No	4723 (81.01%)	1212 (73.19%)	
Total energy, kcal/day			
Mean ± SE	1989.20 ± 793.27	1988.84 ± 697.88	.987
Diabetes			.002
Yes	1197 (20.53%)	276 (16.67%)	
Borderline diabetes	518 (8.89%)	151 (9.12%)	
No	4115 (70.58%)	1229 (74.21%)	
Hypertension			<.001
Yes	2871 (49.25%)	702 (42.39%)	
No	2959 (50.75%)	954 (57.61%)	
Asthma			.413
Yes	838 (14.39%)	225 (13.60%)	
No	4985 (85.61%)	1430 (86.40%)	
COPD			<.001
Yes	460 (7.89%)	85 (5.13%)	
No	5370 (92.11%)	1571 (94.87%)	

Abbreviations: BMI, body mass index; COPD, chronic obstructive pulmonary disease; NHANES, National Health and Nutrition Examination Survey.

### 3.2 Associations between probiotics, prebiotics, or yogurt consumption and COPD prevalence

This study evaluated the effects of probiotics, prebiotics, or yogurt consumption on the prevalence of COPD by multivariate logistic regression models with adjusted covariates (Table 2). The results showed a 25% reduced occurrence of COPD among participants consuming probiotics, prebiotics, or yogurt compared to those who did not. Probiotics, prebiotics, or yogurt consumption was associated with a reduced prevalence of COPD, even after adjusting for gender, education, age, race, marital status, BMI, drinking, smoking, moderate‐intensity activities, vigorous activities, hypertension, asthma, and diabetes (Model 1: OR, 0.68, 95% CI, 0.53–0.87; Model 2: OR, 0.77, 95% CI, 0.59–0.99; Model 3: OR, 0.75, 95% CI, 0.57–0.98).

**TABLE 2:** Associations between probiotics, prebiotics, or yogurt consumption with the prevalence of COPD.

	Model 1	Model 2	Model 3
	OR (95% CI)	OR (95% CI)	OR (95% CI)
Probiotics, prebiotics, or yogurt consumption	0.68 (0.53, 0.87)	0.77 (0.59, 0.99)	0.75 (0.57, 0.98)

*Note*: Model 1 adjusted for age, gender, race, education, and marital status; Model 2 adjusted for age, gender, race, education, marital status, body mass index, smoking status, alcohol drinking status, moderate‐intensity activities, and vigorous activities; Model 3 adjusted for age, gender, race, education, marital status, body mass index, smoking status, alcohol drinking status, moderate‐intensity activities, vigorous activities, hypertension, asthma, and diabetes.

Abbreviations: CI, confidence interval; COPD, chronic obstructive pulmonary disease; OR, odds ratio.

### 3.3 Stratification analysis

**FIGURE 2:**
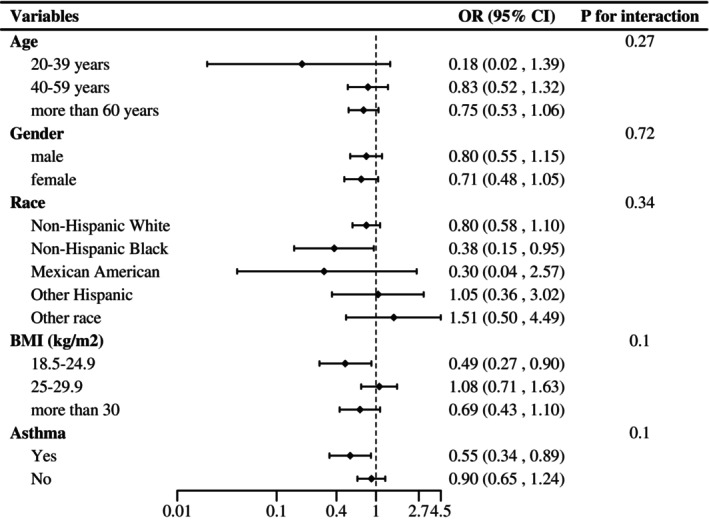
Stratified analyses by age, gender, race, BMI, and asthma of the associations between probiotics, prebiotics, or yogurt consumption with prevalence of COPD. Values adjusted for age, gender, race, education, marital status, body mass index, smoking status, alcohol drinking status, moderate‐intensity activities, vigorous activities, hypertension, asthma, and diabetes.

This study performed stratified analysis based on population characteristics, including age, gender, race, BMI, and asthma, to further investigate the correlations between consuming probiotics, prebiotics, or yogurt and the prevalence of COPD, and to test their interactions (Figure 2). There was a decreasing trend of having COPD for non‐Hispanic black individuals (OR = 0.38, 95% CI, 0.15–0.95), normal‐weight individuals (OR = 0.49, 95% CI, 0.27–0.90), and asthma individuals (OR = 0.55, 95% CI, 0.34–0.89). However, the interaction test showed no significant difference among each stratification group (all *p* interaction >.05). The result of interaction test indicated that there was no significant dependence of age, gender, race, BMI, and asthma on the association between consuming probiotics, prebiotics, or yogurt and the prevalence of COPD.

## 4 DISCUSSION

A comprehensive cross‐sectional study was carried out on a representative group of 7486 individuals in the US, revealing that consuming probiotics, prebiotics, or yogurt was correlated with a 25% reduction in the incidence of COPD. The association is still obtained after controlling potential confounders. The results might suggest that altering intestine microbiota potentially helps to prevent the occurrence of COPD. This study's primary novelty lies in being the inaugural extensive epidemiological study exploring the correlation between consuming probiotics, prebiotics, or yogurt and the prevalence of COPD.

Probiotics are host benefit microorganisms (Akram et al., 2024). Typical probiotics such as *Enterococcus faecium*, *Bacillus*, and *Bifidobacterium* mainly come from probiotic drugs, probiotic food (such as yogurt), and host itself. Prebiotics can stimulate the growth and metabolism of probiotics. The most commonly studied prebiotics include *fructo‐oligosaccharides* (*FOS*), *lactulose*, *isomalto‐oligosaccharides* (*IMO*), *oligosaccharides*, and *xylo‐oligosaccharides* (*XOS*) (Yadav et al., 2022). Other prebiotic food items include yogurt, infant formula, cakes, and cereals. It is well established that probiotics and prebiotics help to control the body's immune response and are employed in treating common respiratory infections like cough, rhinitis, pharyngitis, laryngitis, influenza, and pneumonia. Numerous randomized controlled trials showed that probiotics or prebiotics could reduce the frequency and intensity of respiratory infections, and ameliorate respiratory‐related symptoms (Chong et al., 2019; Luoto et al., 2014; Panigrahi et al., 2017; Wang et al., 2023).

In addition, probiotics can inhibit the rise of *IgE* levels in seasonal allergic disease and reduce allergic manifestations. A meta‐analysis that included 26 randomized controlled trials involving 2644 patients showed that probiotics helped to control nasal symptoms, reduced the nasal symptoms scores, and antiallergic medication use in allergic rhinitis (Wang et al., 2022). Some studies also found that probiotics could improve lung function and clinical symptoms for asthma patients, accompanied by a decrease of cytokines such as interleukin‐6 (*IL‐6*), *IL‐17*, and *IL‐21* (Sadrifar et al., 2023). Du's study found that intake of *Lactobacillus rhamnoses GG* could decrease the incidence of asthma (Du et al., 2019). There is evidence showing that probiotic supplementation helps to improve pulmonary symptoms and pulmonary function, decrease C‐reactive protein (CRP) concentrations and disease severity in sulfur mustard‐exposed COPD patients (Panahi et al., 2017). However, the association of probiotics or prebiotics supplementation with the prevalence of COPD has not been studied in previous studies. Our study showed that intake of probiotics, prebiotics, or yogurt was correlated with a 25% reduction in COPD cases. The result is reliable and is not significantly influenced by gender, age, race, education, BMI, and complications. This evidence demonstrates that probiotics or prebiotics could serve as an alternative therapeutic approach for respiratory diseases.

It remains unclear of the mechanisms for the benefits of probiotics and prebiotics on COPD. The gut–lung axis might influence the association of probiotics with COPD's prevalence. Qu's study indicated that imbalances in intestine microbiota affected the composition of lung microbiota by altering circulating inflammatory cytokines and the movement of intestine microbiota into the lung (Qu et al., 2022). In COPD patients, it was also found a decrease in typical lung microbial diversity, coupled with an increase in detrimental microbiota caused by drug misuse and infections (Ananya et al., 2021). Adding probiotics can directly regulate the dysbiosis of intestine microbiota, and further restore the normal lung microbiota. The normal lung microbiota will inhibit respiratory pathogenic microorganisms and protect COPD individuals from infections and deterioration (Yuksel et al., 2023). Additionally, probiotics have the ability to regulate the pulmonary immune response and potentially reverse the T‐regulatory response in the respiratory tract (Afzaal et al., 2022). Previous studies reported that probiotics could change the disproportion in *Th1/Th2* and *Treg/Th17* immune reactions by reducing *Th2‐* and *Th17‐*driven immune responses (inducing cytokines such as: *IL‐4*, *IL‐6*, *IL‐17*, and *IL‐21*), and enhancing *Th1‐* and *Treg‐*driven immune responses (inducing cytokines such as: *interferon‐γ* and *IL‐10*) (Wang et al., 2022).

Moreover, intake of probiotics might regulate the oxidative unbalance, and decrease the oxidative stress injury of the airways (Roshan et al., 2019). In addition to promote the proliferation of probiotics, some prebiotics also have immunomodulatory effects. For example, prebiotics can prevent the damage from pathogens, enhance the performance of intestinal barrier, and diminish harmful bacteria populations (Parada Venegas et al., 2019). Studies also reported that *oligofructose* supplementation (a kind of prebiotics) helped against oxidative stress, thereby averting the inflammatory responses linked to such stress (Guarino et al., 2020). Therefore, it indicates that probiotics and prebiotics reduce the prevalence of COPD by regulating the inflammatory response.

Current management strategies for COPD include both pharmacological and nonpharmacological strategies. Pharmacological interventions are dominant treatment strategies, which include bronchodilators, anti‐inflammatory agents, and antibiotics (Venkatesan, 2023). These drugs are effective to alleviate symptoms, lessen the regularity and intensity of exacerbations, and enhance the overall health of COPD individuals. However, there is a great concern on the adverse effects of pharmacological therapy, associated with great economic costs. Advantages of probiotics and prebiotics lie in their easy accessibility, low cost, availability in diverse oral forms, well oral tolerance, and safety. Thus, they could serve as good supplementary treatment in managing COPD. It should be cautious to choose suitable products of probiotics, given that the effects of probiotics may vary based on the strains, species, dosage, and length of treatment (Wang et al., 2022).

There are some strengths of our study. Initially, this cross‐sectional study firstly reported the correlation between consuming probiotics, prebiotics, or yogurt and the prevalence of COPD, with a large and representative sample involving 7486 individuals. Hence, it provides specific and reliable evidence for directing the use of probiotics or prebiotics in preventing COPD. Second, an in‐depth discussion was conducted regarding the interactions and mechanisms between probiotics and COPD. However, this study also presents certain limitations. Initially, the cross‐sectional study always fails to uphold a substantial level of causality. Second, the diagnostic criteria of some COPD patients were based on survey data, potentially influencing the model's outcomes. Third, the NHANES evaluated the intake of probiotics or prebiotics, relying on self‐reported data and information from the manufacturers' labels. Forth, due to the limited sample size, subgroup analysis could not be conducted to separately discuss the effects of probiotics, prebiotics, or yogurt on the prevalence of COPD. Fifth, factors such as species, strains, amount, and treatment duration of probiotics or prebiotics were not considered, which might also influence the occurrence of COPD. Lastly, the study observed a decreasing trend of having COPD for non‐Hispanic black individuals, normal‐weight individuals, and asthma individuals, which seemed to suggest that these individuals benefit more from probiotics therapy. However, no significant difference was found after the interaction test. Possible reasons were own to the limited sample size and population variance. Future large‐scale studies which cover populations from different counties should focus on identifying individuals who benefit the most from probiotics therapy to prevent COPD.

## 5 CONCLUSION

This cross‐sectional study found that consuming probiotics, prebiotics, or yogurt had a significant benefit in reducing the prevalence of COPD. The result supports the possibility of gut microbiota modulation using probiotics or prebiotics as an attractive therapeutic target to prevent COPD. However, there are substantial limitations on the design of the study. Cautions are needed when interpreting the result. Future studies should normalize the methodology to overcome these limitations and focus on the separate effects of probiotics, prebiotics, or yogurt on the prevalence of COPD, as well as focus on identifying individuals who benefit the most from probiotics therapy to prevent COPD.

## AUTHOR CONTRIBUTIONS

**Yu Hong:** Conceptualization (lead); formal analysis (lead); investigation (lead); methodology (lead); software (lead); validation (lead); visualization (lead); writing – original draft (lead). **Ting Luo:** Data curation (lead); project administration (lead); resources (lead); supervision (lead); writing – review and editing (lead).

## CONFLICT OF INTEREST STATEMENT

The authors declare that they do not have any conflict of interest.

## Supporting information


Data S1.

## Data Availability

Datasets analyzed in this study can be accessed in the NHANES repository (https://wwwn.cdc.gov/nchs/nhanes/).
