# Supplementary material for: The potential protective effects of probiotics, prebiotics, or yogurt on chronic obstructive pulmonary disease: Results from NHANES 2007–2012
Source: Food Sci Nutr. 2024 Jul 17;12(10):7233–41. doi: 10.1002/fsn3.4332 (PMC11521661; doi:10.1002/fsn3.4332)
Supplement: Supplementary file 1 — Data S1. [file FSN3-12-7233-s001.pdf]

Supplementary Table 1. Search Terms Used to Identify Dietary Supplements or Prescription Drugs That Were Either Labeled With or Contained Ingredients Labeled as Prebiotics or Probiotics in NHANES

Prebiotic search terms	“ACADIA GUM”, “CHICOR”, “GLUCAN”, “GUM ARABIC, “INULIN”, “LACTULOSE”, “OLIGOFRUC”, “OLIGOSAC”, “POLCYDEXTROSE”, “PREBIOTIC”, “PRE-BIOTIC”, “PRE BIOTIC”, “PSYLLIUM”, “RESISTANT STARCH”, “WHEAT DEXTRIN”
Probiotic search terms	“ACIDOPHILUS”, “ANIMALIS”, “BACILLUS”, “BACILLI”, ” BIFIDOBACTERI”, “BIFIDUM”, “BOULARDII”, “BREVE”, “BREVIS”, “BUCHNERI”, “BULGARICUS”, “BUTYRICUM”, “CASEI”, “CAUCASICUS”, “CEREVISIAE”, “CLAUSII”, ” CLOSTRIDI”, “COAGULANS”, “CORYNIFORMIS”, “CRISPATUS”, “DELBRUECKII”, “ESCHERICH”, “E. COLI”, “ECOLI”, “E COLI”, “ENTEROCOCCUS”, “FAECALIS”, “FAECIUM”, “FERMENTUM”, “FLORENTINUS”, “GASSERI”, “HELVETICUS”, “INFANTIS”, “JOHNSONII”, “LACTIS”, “LACTIC ACID BACTERIA”, “LACTOBACILL”, “LACTOCOCCUS”, “LEICHMANNII”, “LEUCONOSTOC”, “LICHENIFORMIS”, “LONGUM”, “MESENTERIC”, “MITIS”, “NISSLE”, “OLIGONITROPHILUS”, “ORALIS”, “PARACASEI”, “PEDIOCOCCUS”, “PLANTARUM”, “PROBIOTIC”, “PRO-BIOTIC”, “PRO BIOTIC”, “PROPIONIBACTERI”, “RATTUS”, “REUTERI”, “RHAMNOSUS”, SACCHAROMYC”, “SALIVARIUS”, “SANGUIS”, “STEAROTHERMOPHILUS”, “STREPTOCOCCUS”, “SUBTILIS”, “THERMOPHILUS”
